# High expression of miR-214 is associated with a worse disease-specific survival of the triple-negative breast cancer patients

**DOI:** 10.1186/s13053-015-0028-z

**Published:** 2015-02-08

**Authors:** Dagnija Kalniete, Miki Nakazawa-Miklaševiča, Ilze Štrumfa, Arnis Āboliņš, Arvīds Irmejs, Jānis Gardovskis, Edvīns Miklaševičs

**Affiliations:** 1 Institute of Oncology, Riga Stradins University, Dzirciema Street 16, Riga, LV-1007 Latvia; 2 Breast Disease Center, Pauls Stradins Clinical University Hospital, Pilsonu Street 13, Riga, LV-1002 Latvia

**Keywords:** MiRNA expressionm, Hereditary and sporadic breast cancers, Triple-negative subtype

## Abstract

**Background:**

Hereditary triple-negative breast cancer patients have better recurrence-free survival than triple-negative sporadic ones. High expression of some of the miRNAs is related to worse overall and disease-free survival of triple-negative breast cancer patients. The attempt to associate expression level of some miRNA in triple-negative hereditary and sporadic breast cancers to disease specific survival was performed in this study.

**Material and methods:**

Study group was made of 18 triple-negative breast cancer patients harboring the *BRCA1* gene mutations and 32 triple-negative sporadic breast cancer patients. Quantitative amount of mir-10b, mir-21, mir-29a, mir-31, and mir-214 by real-time PCR was assessed. The disease-specific survival in relation of high and low levels of some of the miRNAs was analyzed using Log-rank (Mantel-Cox) test.

**Results:**

MiR-214 showed significantly higher expression level in sporadic tissues than in hereditary ones (p = 0.0005). Triple-negative breast cancer patients with high level of miR-214 showed significantly worse disease-specific survival than patients with low level (p = 0.0314).

**Conclusions:**

Our finding suggests that miR-214 possibly could be used as a potential prognostic biomarker for triple-negative breast cancer patients.

**Electronic supplementary material:**

The online version of this article (doi:10.1186/s13053-015-0028-z) contains supplementary material, which is available to authorized users.

## Background

Breast cancer is the most wide spread tumor among women worldwide. In the year 2008 approximately 1.38 million new cases were diagnosed, and 458,400 females died from this malignancy [1]. The majority of these tumors are sporadic while five to 10% are hereditary, and significant proportion of them is due to the inherited mutations either in the *BRCA1* or *BRCA2* gene [2],[3]. There is a strong correlation between the presence of mutations in the *BRCA1/2* gene and morphology of the breast cancer. The triple-negative (TN) morphology of breast tumor is found in the 57% of patients with the *BRCA1* gene mutations and 23% of patients with the *BRCA2* gene mutations [4]. TN breast cancers are referred to estrogen receptor (ER) negative, progesterone receptor (PR) negative, and human epidermal growth factor receptor (HER2) negative tumors and they have tendency to be more aggressive than other subtypes [5],[6]. TN breast cancer patients harboring the *BRCA1* mutations at the time of the diagnosis are younger, have smaller tumor size, and have significantly better recurrence-free and disease-specific survival than TN breast cancer patients with no mutations in the *BRCA1* gene [7]-[10].

MiRNAs are non-coding small RNA molecules that have the ability to regulate gene expression post-transcriptionally, and are involved in the cell differentiation, growth, and apoptosis [11],[12]. In tumors, miRNA expression is changed, and there is a correlation between the changed expression and clinical features of the disease [13],[14]. MiRNAs can act either as tumor inducers or tumor suppressors, and either are up-regulated or down-regulated [15]. One of the most studied tumor inducing miRNA which consistently is up-regulated in different types of malignancies, including breast cancer, is miR-21 [14],[16],[17]. By inhibiting the tumor suppressor tropomyosin-1 gene (*TPM1*) and programmed cell death gene-4 (*PDCD4*), it is directly involved in the growth, proliferation, and invasion of the tumor cells [18],[19]. Another target of miR-21 is phosphatase and tensin homolog gene (*PTEN*) that is involved in the PI3K/Akt pathway [20]-[22]. In glioblastoma cells, miR-21 targets genes that are involved in the major cancer suppression pathways: in p53 pathway (*JMY*, *TOPORS*, *IGFBP3*, *TP53PP2*, *DAXX*, *HNRPK*, and *TP73L*), in TGF-β pathway (*TGFFBR2/3*, *BHPK2*, and *DAXX*), in mitochondrial apoptotic pathway (*APAF1* and *PPIF*) [23]. In breast cancer, the up-regulation of this miRNA is associated with later clinical stage, higher proliferation index Ki-67, and poor prognosis for the patient [14],[17]. Breast cancer patients with tumor size more than two centimeters have significantly higher expression of miR-21 compared to those with tumor size less than two centimeters [24]. Another oncogenic miRNA which over-expression is associated with breast cancer is miR-10b. MiR-10b is involved in the Rhoc-Akt signaling pathway; Rhoc-Akt pathway is suppressed by the repression of HOXD10 thus promoting cancer cell invasion [25]. Moreover, breast cancer patients with tumor size greater than two centimeters have higher miR-10b expression than those with tumor size less than two centimeters [24]. In ovary cancers, miR-214 is expressed differently between the carriers of the *BRCA1* gene mutations and non-carriers; miR-214 is down-regulated in the patients with mutations in the *BRCA1* gene [26]. One of the targets that miR-214 regulate is *PTEN* gene; in the *EGFR* mutated non-small lung tumor cell lines up-regulated miR-214 through the PTEN/AKT pathway induces resistance to gefitinib [27].

Some of the miRNAs have shown different expressions not only within the specific subtype of breast cancer but among distinct subtypes of breast cancer, as well. MiR-210 is differently expressed between TN and ER positive/HER2 negative breast cancers: higher expression is in TN breast cancers than in ER positive/HER2 negative breast cancers [28]. A non-significant tendency of high expression level of miR-210 and other miRNAs (miR-21, miR-221, and miR-222) is related to worse overall and disease-free survival of TN breast cancer patients [29]. In another study, TN patients with high expression of miR-210 showed considerably worse overall and disease-free survival than those with low expression of miR-210 [28]. In contrast, breast cancer patients with negative expression of miR-34b have worse overall and disease-free survival compared to those with positive expression of miR-34b [30].

The aim of this study was to look for the miRNA that differs in expression between TN hereditary and sporadic tumors and associate expression level of some miRNA to disease specific survival of TN breast cancer patients.

## Material and methods

Eighteen TN breast cancer patients harboring the *BRCA1* gene mutations and 32 triple-negative sporadic breast cancer patients hospitalized at Pauls Stradins Clinical University Hospital and/or Latvian Oncology Center from 2004 to 2011 were involved in this study. All patients signed informed consent forms.

The *BRCA1* gene mutations (5382insC, 4153delA, and C61G) were determined by multiplex polymerase chain reaction [31]. Breast cancer patients with the *BRCA1* gene mutations were defined as hereditary [31].

TN tumors were defined as ER and PR 0% and HER2 protein 0%. This study included only those TN tumor tissues which tumor cell content per specimen was more than 50%. All histological data was reviewed and evaluated by pathologist. Immunohistochemistry data was analyzed and interpreted by pathologist.

MiRNAs were isolated from the formalin-fixed and paraffin embedded tumor tissues with the RecoverAll Total Nucleic Acid Isolation Kit (Ambion, Applied Biosystems). Reaction of the reverse transcription was done with the TaqMan MicroRNA Reverse Transcription Kit (Applied Biosystems) on the TProfessional Thermal Cycler (Biometra). Quantitative analysis of miRNAs was performed with real-time PCR (Rotor-Gene 6000, Corbett) using TaqMan MicroRNA Assays (Applied Biosystems). Each sample was performed in three repeats. The expression levels were analyzed with the Rotor-Gene Q Series Software 1.7 using comparative quantitation analysis. Each miRNA was normalized by the internal reference RNU6B.

The disease-specific survival was evaluated from the date of the diagnosis till the date of the death from the malignancy. The disease-specific survival was analyzed using the Log-rank (Mantel-Cox) test. The level of the statistical significance was set at the 95%. The median follow-up period of the TN breast cancer patients was 40 months. According to the American Joint Committee on Cancer (AJCC), as a T_1_/T_2_ group were defined tumors that were ≤ 20 mm to ≤ 50 mm in dimension. T_3_/T_4_ group were defined tumors that were >50 mm in dimension to any size with direct extension to the chest wall and/or to the skin [AJCC]. Whitney–Mann test was used to calculate miRNA expression differences between TN-H and TN-S tumor tissues. Clinical and pathological characteristics between the *BRCA1* gene mutation carriers and non-carriers were compared by *t*-test and Fisher’s exact test.

## Results

TN breast cancer patients with the *BRCA1* gene mutations at the time of the diagnosis were younger than sporadic individuals (p = 0.0648). Higher frequency of TN patients with staging T_3_ were in sporadic group than in hereditary one (p = 0.0731). Between hereditary breast cancer patients stage I was more frequent than between sporadic ones (p = 0.0889). Medullary carcinomas were significantly more in hereditary group comparing to sporadic one (p = 0.0442) (Table 1).

**Table 1:** Clinical and pathological characteristics of*BRCA1*mutation carriers (n = 18) and non-carriers (n = 32)

Characteristics	*BRCA1*mutation carriers, n (%)	*BRCA1*mutation non-carriers, n (%)	P-value
Age at diagnosis, years			
Median	46	55	0.0648
Range	27-72	28-78	
T stage			
T_1_	7 (38.89)	6 (18.75)	0.1797
T_2_	7 (38.89)	15 (46.88)	0.7676
T_3_	1 (5.56)	9 (28.13)	0.0731
T_4_	3 (16.67)	2 (6.25)	0.3363
Nodal status			
N_0_	12 (66.67)	19 (59.38)	0.7637
N_1_	0 (0.00)	4 (12.50)	0.2828
N_2_	5 (27.28)	6 (18.75)	0.4944
N_3_	1 (5.56)	3 (9.38)	0.9999
Metastasis			
M_0_	17 (94.44)	31 (96.88)	0.9999
M_1_	1 (5.56)	1 (3.13)	0.9999
Stage			
I	7 (38.89)	5 (15.63)	0.0889
II	5 (27.78)	14 (43.75)	0.3659
III	5 (27.78)	12 (37.50)	0.5482
IV	1 (5.56)	1 (3.13)	0.9999
Histology			
Ductal carcinoma	14 (77.78)	28 (87.50)	0.3984
Tubular carcinoma	0 (0.00)	1 (3.13)	0.9999
Medullary carcinoma	3 (16.67)	0 (0.00)	0.0442
Papillary carcinoma	1 (5.56)	2 (6.25)	0.9999
No data	0 (0.00)	1 (3.13)	
Tumor grade			
Moderately differentiated	3 (16.67)	1 (3.13)	0.1142
Poorly differentiated	12 (66.67)	27 (84.38)	0.1142
No data	3 (16.67)	4 (12.50)	
Ki-67	73	70	0.6494

Association between the median ± standard deviation expression of each miRNA and T stages (T_1/2_ and T_3/4_) was explored. Statistically not quite a significant difference between T_1/2_ and T_3/4_ groups in the case of miR-31 was observed (p = 0.0666) (Table 2).

**Table 2:** Association between expression level of each miRNA and T stage

miRNA	T_1/2±SD_	T_3/4±SD_	P-value
miR-10b	0.289 ± 0.288	0.421 ± 0.210	0.1411
miR-21	6.180 ± 5.736	7.910 ± 4.554	0.3093
miR-29a	1.475 ± 1.089	1.825 ± 0.983	0.3399
miR-31	0.299 ± 0.493	0.592 ± 0.472	0.0666
miR-214	1.230 ± 1.95	1.700 ± 0.856	0.3775

**Figure 1:**
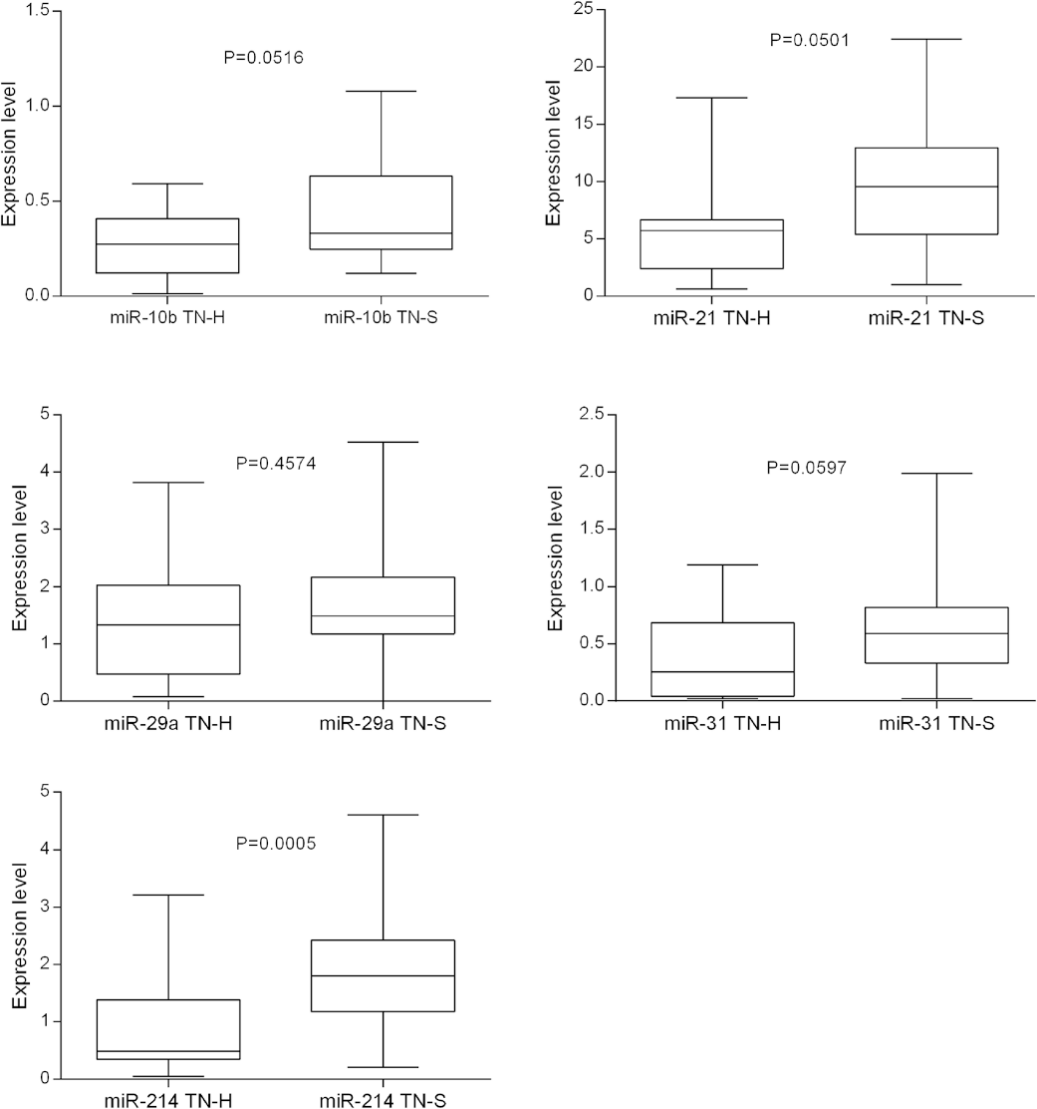
**Different miRNA expression between TN-H and TN-S breast cancer tissues.** Statistical significance was set at the 95% level.

The expression level of miR-10b, miR-21, miR-29a, miR-31, and miR-214 in 18 TN-H and 32 TN-S breast tumors was analyzed. Measurements were normalized to internal control RNU6B. After normalization outliers were excluded. The median expression level ± interquartile range (Q1; Q3) of miR-10b, miR-21, miR-29a, miR-31, and miR-214 in TN-H cancer tissues was 0.275 ± 0.287 (0.122; 0.408), 5.725 ± 4.250 (2.408; 6.658), 1.330 ± 1.552 (0.478; 2.030), 0.254 ± 0.642 (0.041; 0.684), and 0.489 ± 1.027 (0.350; 1.378), respectively. The median expression level ± interquartile range (Q1; Q3) of miR-10b, miR-21, miR-29a, miR-31, and miR-214 in TN-S breast cancer tissues was 0.330 ± 0.381 (0.249; 0.631), 9.580 ± 7.545 (5.405; 12.950), 1.490 ± 0.990 (1.180; 2.170), 0.592 ± 0.487 (0.332; 0.819), and 1.800 ± 1.250 (1.170; 2.420), respectively. MiR-214 showed significantly higher expression level in TN-S tumor tissues than in TN-H ones (Figure 1).

**Figure 2:**
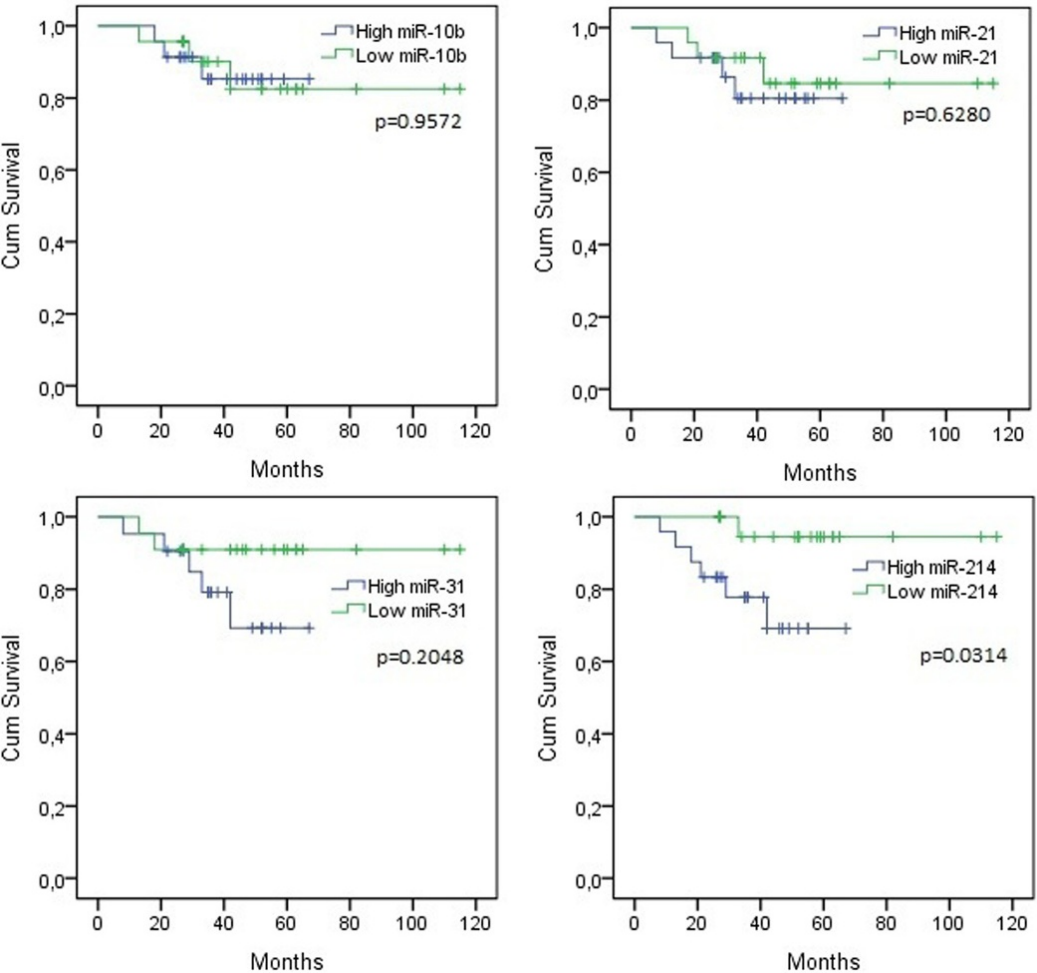
**Disease-specific survivals of TN breast cancer patients in regard of high and low expression of miRNAs.** Statistical significance was set at the 95% level.

Disease-specific survival of the TN breast cancer patients in respect of high and low levels of miR-10b, miR-21, miR-31, and miR-214 was analyzed. High and low expression levels were defined as values above and below median expression level, respectively. The median expression ± interquartile range (Q1; Q3) of miR-10b, miR-21, miR-31, and miR-214 was 0.327 ± 0.337 (0.198; 0.535), 6.990 ± 9.245 (3.105; 12.350), 0.488 ± 0.599 (0.214; 0.813), and 1.455 ± 1.593 (0.625; 2.218), respectively. TN breast cancer patients with high expression level of miR-214 showed significantly worse disease-free survival than patients with low expression of this miRNA (Figure 2).

## Discussion

In this study, the *BRCA1* gene mutation carriers at the time of the diagnosis were younger than patients with no evadable changes in the *BRCA1* gene. Finding is consistent with other studies; the *BRCA1* gene mutation carriers at the time of the diagnosis were younger than sporadic breast cancer patients [8]. Medullary carcinomas were seen more in *BRCA1* carriers than non-carriers. There has been observed a weak correlation between *BRCA1* mutation status and medullar histology of breast cancer [32],[33].

TN sporadic breast cancer tissues showed significantly higher expression level of miR-214 than TN hereditary individuals that are consistent with the finding in other study; miR-214 is expressed differentially in ovary cancer patients with and without the *BRCA1* gene mutations [26]. High grade serous carcinoma patients with any loss within the *BRCA1* gene show lower expression of miR-214 than patients with no change [26]. The disease-specific survival in respect of high and low expression level of miR-214 was analyzed. TN breast cancer patients with high expression level of miR-214 have significantly worse disease-specific survival than patients with low expression of miR-214. According to the results of this study, in the breast cancer, miR-214 may act similarly as oncogene that is consistent with the finding in other study. MiR-214 is up-regulated in preoperative serum samples of breast cancer patients; whereas, in post-operative serum samples, it is decreased and increased miR-214 correlates with positive lymph node status [34]. MiR-214 plays an important role not only in the ovary cancer but as well in the breast cancer development. It is not clear how BRCA1 dysfunction can influence the level of miR-214 in ovarian and breast tumors as yet. It is known that miR-214 targets the *PTEN* gene; by targeting *PTEN* Akt pathway is activated thus resulting in the cell survival [35]. In many different types of cancers, in about 40% of ovarian and breast cancers, Akt kinase activity has been detected increased [36].

In advanced (metastatic) breast cancers, expression of miR-10b is up-regulated as compared to the primary ones [36]. MiR-10b is directly involved in the suppression of the *HOXD10* that in turn activates expression of the pro-metastatic gene *RHOC*[36]. MiR-10b correlation between tumor size, histological grade, clinical stage, positive lymph node status, and HER2 expression is positive [37]. While the correlation between high expression of miR-10b and HER2 expression is positive; the correlation between miR-10b expression and PR and ER status is negative [37]. Over-expression of miR-10b* is associated with reduced disease-free, relapse-free, and metastasis-free survivals, compared to those with low expression level [38]. TN-S breast cancer tissues have higher expression level of miR-10b than TN-H ones, though the difference is not quite statistically significant. In addition, the frequency of T_3_ is higher in sporadic group than in hereditary one. The TN hereditary breast cancer patients at the time of diagnosis have smaller tumor size than sporadic ones [7]-[9]. A non-significant higher frequency of T_3_ in sporadic group suggests that sporadic breast cancer patients have higher frequency of tumors that are greater than 5 cm across than hereditary ones. Moreover, the frequency of stage I among hereditary breast cancer patients is higher than between sporadic breast cancer patients. Findings in this study support findings in other studies.

Another miRNA that in this study was up-regulated in TN-S tissues as compared to TN-H ones was miR-21. As well as in this case, difference between groups was not quite statistically significant. MiR-21 is up-regulated in TN primary breast cancers as compared to healthy breast tissues [29]. Expression of miR-21 is significantly higher in ERα positive, ErbB2 negative, and PR positive than in ERα negative, ErbB2 positive, and PR negative breast cancers [16]. MiR-21 is regulated by both ER (ERα and ERβ) receptors. Interaction between estradiol (E2) and one of the two ER receptors leads to the inhibition of miR-21 expression thus resulting in a loss of suppression of PDCD4, PTEN and BCL2 protein expression [39]. In addition, interaction between E2 and ERα directly increases transcription of BCL2 [39]. The mRNA profiling analysis revealed that in the adjacent normal breast tissues compared to TN ones, oncogenic BCL2 is down-regulated whereas miR-21 in TN breast cancer tissues is over-expressed [40]. Breast cancer patients with ER negative and PR negative receptor status have significantly higher expression of miR-21 than breast cancer patients with ER positive and PR positive receptor status [24]. TN breast cancer patients with high expression level of miR-21 have a non-significant tendency of worse overall and disease-free survival than to those with low expression of miR-21 [29].

In this study higher expression level of miR-31 was in TN-S tumor tissues than in TN-H ones; however, as well in this case the difference was not quite statistically significant. Up-regulation of miR-31 is associated with less aggressive breast cancer subtypes, like luminal ones; whereas down-regulation is associated with more aggressive breast cancer subtypes, like triple-negative ones. In the MDA-MB-231 (triple-negative breast cancer subtype) cell lines miR-31 is found down-regulated whereas in the MCF7 (luminal breast cancer subtype) cell lines up-regulated [41],[42].

## Conclusions

According to the results of this study miR-214 is an indicator for the TN breast cancer patient’s poor prognosis and possibly could be used as a potential prognostic biomarker for TN breast cancer subtype.

## Authors’ original submitted files for images

Below are the links to the authors’ original submitted files for images. Authors’ original file for figure 1 Authors’ original file for figure 2
