# Supplementary figures and images for: High expression of miR-214 is associated with a worse disease-specific survival of the triple-negative breast cancer patients
Source: Hered Cancer Clin Pract. 2015 Feb 8;13:7. doi: 10.1186/s13053-015-0028-z (PMC4335782; doi:10.1186/s13053-015-0028-z)

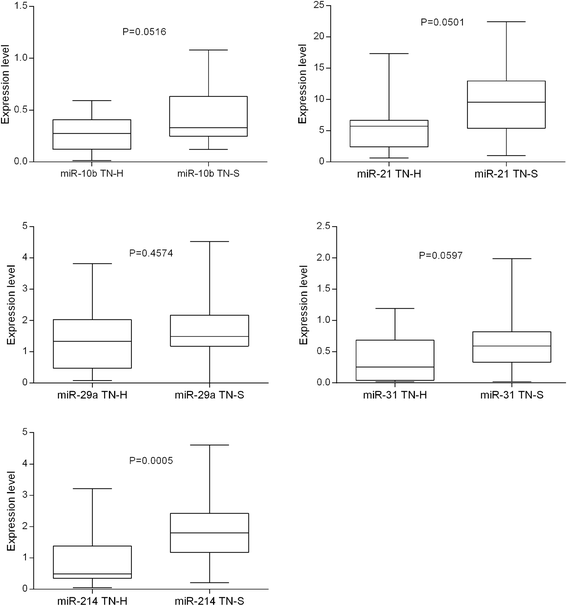

Supplement: Supplementary file 1 — Authors’ original file for figure 1 [file 13053_2015_28_MOESM1_ESM.gif]

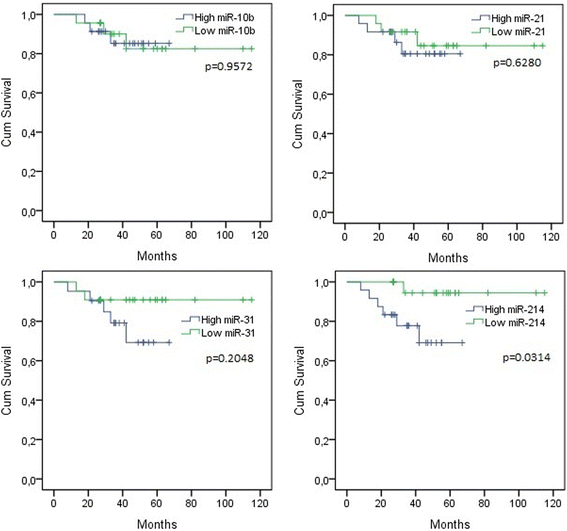

Supplement: Supplementary file 2 — Authors’ original file for figure 2 [file 13053_2015_28_MOESM2_ESM.gif]
